# Comparison of Coopdech®, CoPilot®, Intubrite®, and Macintosh laryngoscopes for tracheal intubation during pediatric cardiopulmonary resuscitation: a randomized, controlled crossover simulation trial

**DOI:** 10.1007/s00431-015-2567-8

**Published:** 2015-05-21

**Authors:** Łukasz Szarpak, Łukasz Czyżewski, Zenon Truszewski, Andrzej Kurowski, Tomasz Gaszyński

**Affiliations:** Department of Emergency Medicine, Medical University of Warsaw, Lindleya 4 Street, 02-005 Warsaw, Poland; Department of Nephrologic Nursing, Medical University of Warsaw, Warsaw, Poland; Department of Anesthesiology, Cardinal Wyszynski National Institute of Cardiology, Warsaw, Poland; Department of Emergency Medicine and Disaster Medicine, Medical University of Lodz, Lodz, Poland

**Keywords:** Intubation, Pediatrics, Laryngoscopes, Training, Randomization

## Abstract

The aim of the study was to compare the intubation times and success rates of various laryngoscopes during resuscitation in pediatric emergency intubation with uninterrupted chest compression on a standardized pediatric manikin model. This was a randomized crossover study with 107 paramedic participants. We compared times to successful intubation, intubation success rates, and glottic visibility using a Cormack–Lehane grade for Macintosh, Intubrite®, Coopdech®, and Copilot® laryngoscopes. One hundred seven paramedics (mean age 31.2 ± 7.5 years) routinely involved in the management of prehospital care participated in this study. Intubation success rates (overall effectiveness), which was the primary study endpoint, were highest for the Coopdech® and CoPilot® devices (100 %) and were lowest for Intubrite® (89.7 %, *p* < *0.001*) and Macintosh (80.4 %, *p* < *0.001*). The secondary study endpoint, time to first effective ventilation, was achieved fastest when using the Coopdech® laryngoscope (21.6 ± 6.2 s) and was significantly slower with all other devices (Intubrite® 25.4 ± 10.5 s, *p* = 0.006; CoPilot® 25.6 ± 7.4 s, *p* = 0.007; Macintosh 29.4 ± 8.2 s, *p* < 0.001).

*Conclusion*: We conclude that in child simulations managed by paramedics, the Coopdech® and Copilot® video laryngoscopes performed better than the standard Macintosh or Intubrite® laryngoscopes for endotracheal intubation during child chest compression.

**Table:** 

**“What is Known”** • *Pediatric intubation performed by paramedics in prehospital conditions using a laryngoscope with Miller or Macintosh blades is varied and ranges from 63.4 to 82 %*.
**“What is New”** • *This work is the first one evaluating mentioned airway devices in pediatric CPR provided by paramedics*. • *The results of this work can influence choice of airway device for clinical use in pediatric CPR*.

## Introduction

The main cause of cardiac arrest in pediatric patients is respiratory failure [11, 14, 23]. Ensuring an adequate airway and adequate oxygenation of the patient is a key element of CPR. The 2010 European Resuscitation Council (ERC) guidelines emphasize that interruptions in chest compression should be minimized during cardiopulmonary resuscitation (CPR) [1]. These guidelines also suggest that the intubator should be able to secure the airway without interrupting chest compression (CC).

There are many reports in the literature regarding the effectiveness of intubation of children in hospital settings [2, 10], in which the intubator—usually the anesthetist—has the appropriate hardware facilities, including a full range of sedative and relaxative drugs. For Emergency Medical Services (EMS) teams, when the paramedic cannot count on the help of an experienced anesthetist, child intubation during CPR can cause many problems. According to Gerritse et al., child intubation effectiveness when performed by paramedics in prehospital settings was insufficient and ranges from 63.4 to 77 % [7, 8, 23]. This means that one in four children requiring airway protection and proper ventilation is not intubated, or the endotracheal tube is inserted incorrectly. Endotracheal intubation, which is currently the gold standard for airway management, is not currently performed only with Macintosh or Miller blades. In “Plan A” of his algorithm for airway management in children, Dr. Philip Ragg [13] indicates the possibility of using video laryngoscopes as a method of intubation.

The aim of the study was to compare time and success rates of different available laryngoscopes for pediatric emergency intubation during resuscitation with uninterrupted chest compression on a standardized pediatric manikin model.

## Methods

This manuscript reports on our randomized controlled trial in accordance with the CONsolidated Standards of Reporting Trials (CONSORT) statement [16]. Approval was granted by the International Institute of Rescue Research (Warsaw, Poland) Institutional Review Board (Approval 11.2014.02.19, November 5th, 2014). Prior to the study, commencing it was registered at the ClinicalTrials register (www.clinicaltrials.gov, identifier NCT02289664).

### Study design

We conducted a randomized crossover trial comparing the effects of the Macintosh, Intubrite®, Coopdech®, and CoPilot® laryngoscopes on intubation parameters including time-to-intubation and number of intubation attempts. With voluntary written, informed consent, 107 paramedics were recruited that satisfied the following inclusion criteria: (1) they had not performed more than 50 clinical child intubations by direct laryngoscopy, and (2) they had not received any training in endotracheal intubation using the Intubrite®, Coopdech®, and CoPilot® devices prior to the study.

**Fig. 1:**
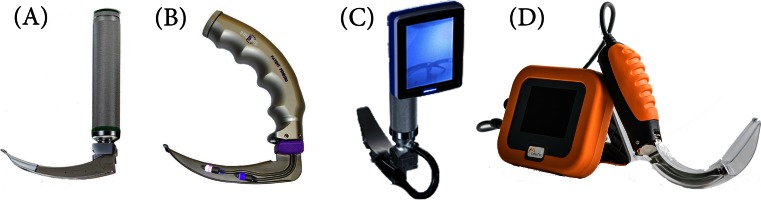
Laryngoscopes used for this study were **a** standard Macintosh laryngoscope, **b** Intubrite® laryngoscope, **c** (3) Coopdech® video laryngoscope portable VLP-100, and **d** (4) CoPilot® video laryngoscope

The devices used for the study were (Fig. 1):

1. Standard Macintosh laryngoscope, blade 2 (Macintosh) (HEINE Optotechnik, Munich, Germany).
2. Intubrite® with Macintosh #2 blade (Intubrite®) (Intubrite®, LLC; Vista, CA, USA).
3. Coopdech® Video Laryngoscope Portable VLP-100 (Coopdech®) (Daiken Medical CO., LTD.; Osaka, Japan).
4. CoPilot® Video Laryngoscope (CoPilot®) (Magaw Medical; Fort Worth, TX, USA).

All intubations were performed using a Magill tracheal tube with 5.0-mm internal diameter (ID). Lubricant was already applied to the tracheal tube, and a 10-mL syringe to block the tube’s cuff, as well as an Ambu® resuscitator bag (Ambu, Copenhagen, Denmark), was readily available and within range of the participant.

Prior to the trial, all participants were given a 30-min standardized training session and a 10-min practice session. The standardized training session consisted of a PowerPoint (Microsoft, Redmond, WA, USA) presentation with voice-over and a series of technique videos. The PowerPoint presentation reviewed airway anatomy, features of the laryngoscopes, and study protocol, while the technique videos demonstrated the use of all three laryngoscopes. The participants were given 10 min to practice with the three laryngoscopes on a manikin until they were comfortable with the devices; then, each participant had a maximum of three successful intubation attempts per laryngoscope. The manikin head used in the practice session and timed trials was a PediaSIM CPR training manikin (FCAE HealthCare, Sarasota, FL, USA), which is designed to be an accurate representation of a six-year-old child. Chest compression was performed using LUCAS-2 (Physio-Control, Redmond, WA, USA). Participants were allowed to ask questions at any time during the practice session and constructive feedback was provided by an instructor.

**Fig. 2:**
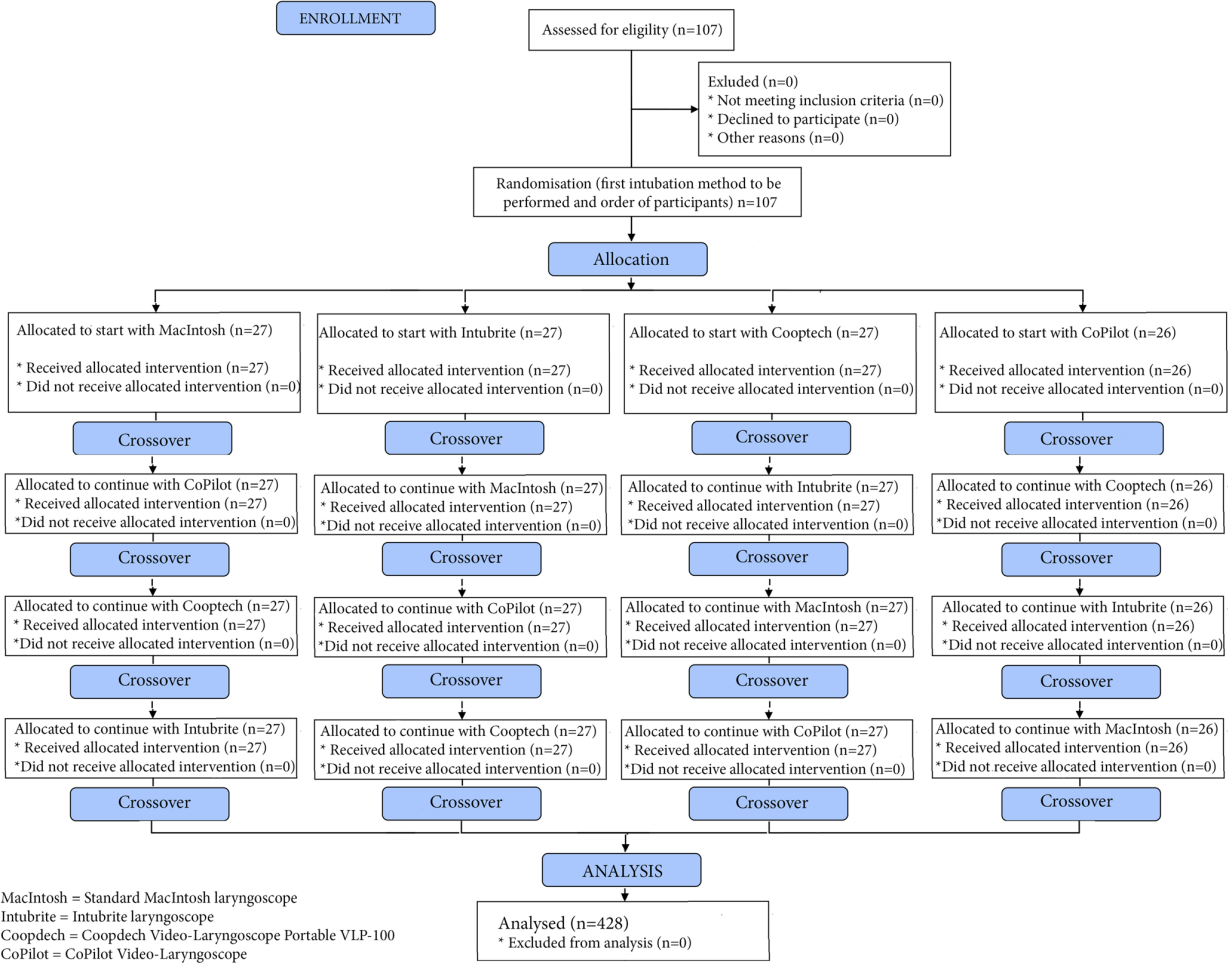
Flow chart of design and recruitment of participants according to CONSORT statement

A Research Randomizer program was used [www.randomizer.com] to divide the volunteers into four groups and to determine the order in which to apply the different endotracheal intubation (ETI) devices within each group. The first group attempted ETI using the Macintosh laryngoscope, the second using the Intubrite®, the third using the Coopdech®, and the fourth using the CoPilot® (Fig. 2). After completing the ETI procedure, participants had a 30-min break before performing intubation using another laryngoscope.

### Measurements

The primary endpoint of the study was the success rate of intubation. The secondary endpoint was defined as the time from insertion of the blade between the teeth to the first manual ventilation of the manikin’s lungs. If the examinee failed at all attempts, the case was excluded from the time calculations. After each attempt, participants were asked to rate the glottic view they had during the attempt using a Cormack–Lehane grade [4]. Quantitative data are presented as mean and standard deviation.

### Statistical analysis

The R statistical package for Windows (version 3.0.0) was used for statistical analysis. Results were reported as mean and standard deviation (±SD) or absolute numbers and percentages. The Kolmogorov–Smirnov test was used to assess the normality of the distributions. As data were found not to be normally distributed, non-parametric tests were applied. We used a median test for continuous variables and an uncertainty coefficient test for categorical data. The cumulative success rate associated with time to complete tracheal intubation was analyzed using Kaplan–Meier analysis. *p* value <0.05 was considered to be statistically significant.

## Results

### Study collective

One hundred and seven paramedics (42 female, 39.3 %) participated in this study. They each had 1 to 2 years of clinical experience and had performed about 50 tracheal intubations each. None had previously used Coopdech®, CoPilot®, or Intubrite® laryngoscopes. No complications such as dental compression were noted in the trial. No data were excluded from analysis. Seventy-three paramedics (27 female, 36.9 %) worked in teams of emergency medical services (EMS), 34 (15 female, 44.1 %) in hospital emergency units. Mean age was 31.2 ± 7.5 years. Average experience of clinical children intubation was 27 ± 5 intubations.

### Success rate

The success rate after the first attempt using the Macintosh, Intubrite®, Coopdech®, and CoPilot® laryngoscopes varied and amounted to 58.9 vs. 69.1 % vs. 100 vs. 100 %. The overall effectiveness of intubation is presented in Table 1. There was a statistically significant difference in intubation success rate between Macintosh and Intubrite® (*p* = 0.019), as well as Coopdech® (*p* < 0.001) and CoPilot® (*p* < 0.001). There was also a statistically significant difference between Intubrite® and Coopdech® (*p* < 0.001) and CoPilot® (*p* < 0.001). Among the four analyzed laryngoscopes, intubation was most effective with the Coopdech® and Copilot® laryngoscopes.

**Table 1:** Time to and success of intubation

Type of laryngoscope blade	Time to intubation (s) [mean (SD)]	Tracheal intubation attempts			
		First (%)	Second (%)	Third (%)	Failed (%)
Macintosh	29.4 ± 8.2	58.9	79.4	80.4	19.6
Intubrite	25.4 ± 10.5	69.1	85.0	89.7	10.3
Coopdech	21.6 ± 6.2	100	100	100	0.0
CoPilot	25.6 ± 7.4	100	100	100	0.0

*Macintosh* standard Macintosh laryngoscope, *Intubrite* Intubrite® laryngoscope, *Coopdech* Coopdech® video laryngoscope portable VLP-100, *CoPilot* CoPilot® video laryngoscope

### Time to first ventilation

**Fig. 3:**
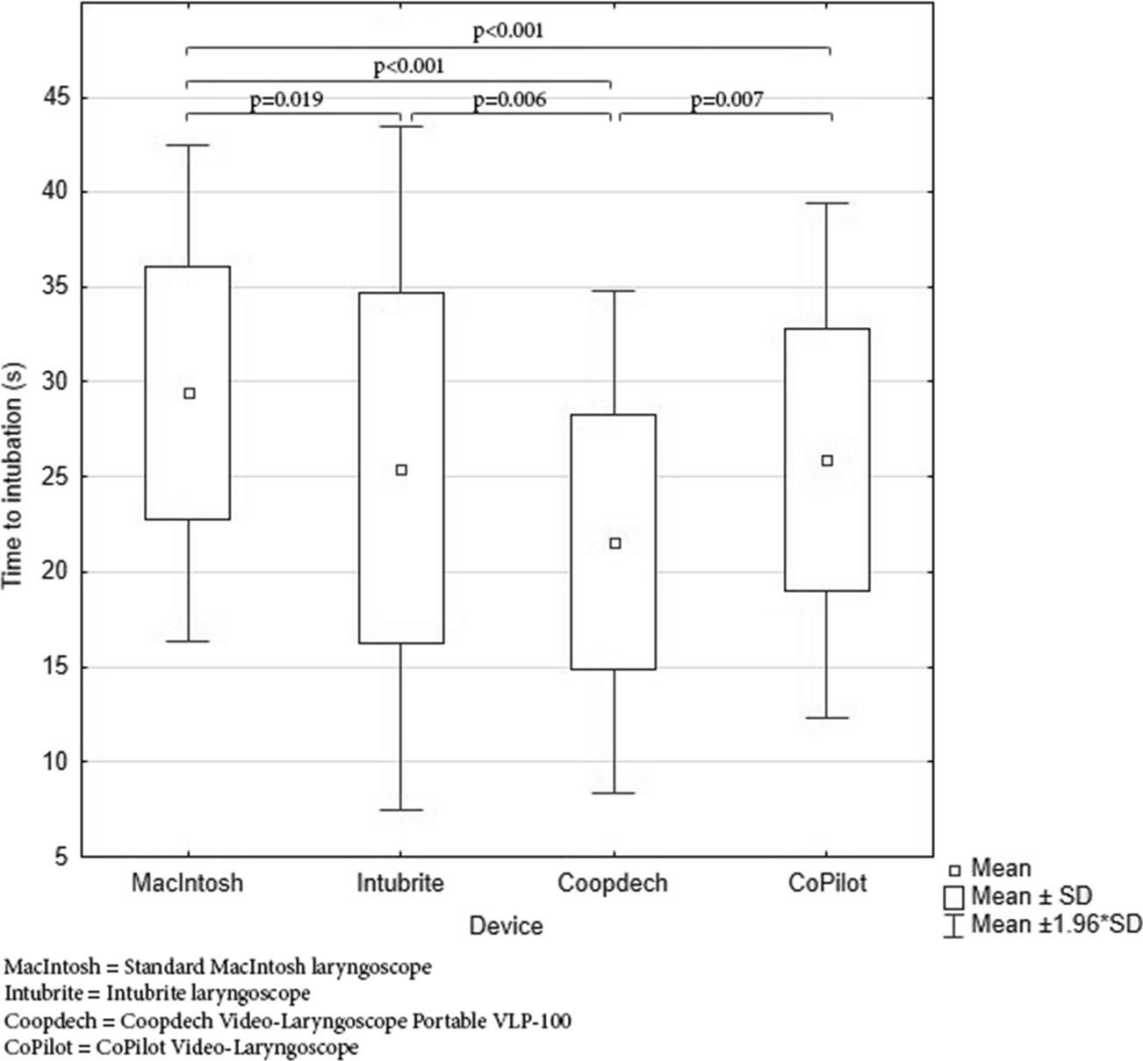
Time required for tracheal intubation with four types of laryngoscopes. *MacIntosh* standard Macintosh laryngoscope, *Intubrite* Intubrite® laryngoscope, *Coopdech* Coopdech® video laryngoscope portable VLP-100, *CoPilot* CoPilot® video laryngoscope

The average times to successful intubation using Macintosh, Intubrite®, Coopdech®, and Copilot® are presented in Fig. 3. Analysis showed that the shortest average time of child intubation during uninterrupted chest compressions was achieved when using Coopdech® (21.6 ± 6.2 s), and the longest when using Macintosh (29.4 ± 8.2 s). A statistically significant difference was noticed between Coopdech® and Macintosh (*p* < 0.001) and Coopdech® and CoPilot® (*p* = 0.007) as well as between Coopdech® and Intubrite® (*p* = 0.006). A statistically significant difference was also observed between Intubrite® and Macintosh (*p* = 0.019) and between CoPilot® and Macintosh (*p* < 0.001).

### Quality of glottic view

Glottic view quality was best with Coopdech® and CoPilot®, where 100 % reported a quality of glottic view corresponding to a Cormack–Lehane classification of I (Table 2).

**Table 2:** Grade of glottic view according to the Cormack–Lehane grading that was achieved with the different ETI devices

Device	C/L I	C/L II	C/L III	C/L IV
Macintosh	78 (72.9 %)	26 (24.3 %)	3 (2.8 %)	0 (0.0 %)
Intubrite®	84 (78.5 %)	23 (11.5 %)	0 (0.0 %)	0 (0.0 %)
Coopdech®	107 (100 %)	0 (0.0 %)	0 (0.0 %)	0 (0.0 %)
CoPilot®	107 (100 %)	0 (0.0 %)	0 (0.0 %)	0 (0.0 %)

Data is given in absolute numbers and percentage

## Discussion

The main cause of sudden cardiac arrest in children is respiratory failure and not heart disease as is the case with adults [1, 11, 14, 23]. The 2010 European Resuscitation Council (ERC) and American Heart Association (AHA) resuscitation guidelines emphasize the importance of minimizing interruptions to chest compression during cardiopulmonary resuscitation (CPR) [4, 17, 23]. These guidelines also suggest that the intubator should be able to secure the airway without interrupting chest compression. However, direct laryngoscopy and tracheal intubation during emergencies remain a challenge to medical practitioners who do not have clinical experience with the techniques or who are expected to perform intubation in difficult situations.

In the present study conducted under simulated resuscitation, intubation efficiency with Macintosh was 80.4 % and 89.7 % with Intubrite®. The higher efficiency of Intubrite® may be due to the different profiles of its handle and better lighting placement on the laryngoscope blade. The effectiveness of direct intubation on children using a laryngoscope with Macintosh or Miller blades performed by paramedics in out-of-hospital conditions is varied and ranges from 63.4 to 77 % in the study by Gerritse et al. [7, 8], 79.8 % in the study by Tollefsen et al. [19], and 82 % in the study by Vilke et al. [20]. Research indicates that low first attempt efficiency of intubation of children does not apply only to intubation performed by paramedics and activities under prehospital care. The study performed in 13 emergency departments by Choi et al. indicates that the first intubation attempt effectiveness with laryngoscope with Macintosh or Miller blades, for doctors with a specialization in emergency medicine, was 74.4 and 50 % for people with other specialties [3]. The poor efficiency of the first intubation attempts performed by paramedics and emergency physicians is also indicated in a study by Ehrlich et al. [5], in which the first intubation attempt effectiveness was 45 %, when performed by paramedics and 67 % [5] for doctors with emergency medicine specialization outside trauma centers. Eich et al. also showed in their study that intubation effectiveness increases with the age of the child. In prospective studies examining prehospital intubation of children by doctors with a specialization in the field of emergency medicine, the first intubation attempt effectiveness through the mouth was varied and was 53.9 % for infants, 68.2 % for children under 5 years of age, and 95.7 for children aged 6–14 years [6].

Alternative methods of airway management may be useful, especially for those who do not have regular exposure to situations in which airway management skills are required. In these situations, it is vital that an effective airway can be obtained quickly and without extensive prior training using the airway device. The authors of several studies indicate the higher efficiency of video laryngoscopes on direct laryngoscopy child intubation [9, 18, 21, 22].

The main findings of the study are that video laryngoscopes (Coopdech® and CoPilot®) may provide benefits regarding time to final placement compared to direct laryngoscopy. There is no significant difference in time to being operational in the case of those laryngoscopes. The first intubation attempt effectiveness using Coopdech® and CoPilot® in our study was 100 % for both devices. Average intubation time was shorter when using Coopdech® (21.6 ± 6.2 s) than using CoPilot® (25.6 ± 7.4 s.).

This study is the first study showing intubation effectiveness on manikins resembling a six-year-old child using the CoPilot® and Coopdech® video laryngoscopes. Saito et al. evaluated the intubation effectiveness of Coopdech® VLP-100 under simulated difficult airways. He found vocal cord visibility and intubation efficiency to be better with the Coopdech® than with the Macintosh laryngoscope [15].

Video laryngoscopes are supposed to help during intubation. As demonstrated in the study, the effectiveness of pediatric intubation while using the Coopdech® and CoPilot® laryngoscopes was 100 %. These devices offered the most effective “method for pediatric intubation during resuscitation” out of all the tested ETI devices. Analysis showed that according to the researched paramedics, video laryngoscopes provided a better view of the larynx area.

This study has a number of significant limitations. Firstly, it is a manikin study and does not involve real patients, and a recognized problem with manikin studies is that the times required to perform airway interventions are generally quicker than in patients. However, according to the International Liaison Committee on Resuscitation (ILCOR), randomized clinical trials for cases of cardiac arrest are unethical and cannot determine the expected benefits of CPR [12]. In both video laryngoscope and optical laryngoscope (e.g., AirTraq), reduce hampering their use may be the presence of fluids (including blood) in the oral cavity which reduces or completely prevents the visibility of the glottis. The strengths of this study include the use of a highly advanced patient simulator for performing pediatric advanced life support and the randomized crossover procedure.

## Conclusions

We conclude that in child simulations managed by paramedics, the Coopdech® and Copilot® video laryngoscopes performed better than the standard Macintosh or Intubrite® laryngoscopes for endotracheal intubation during child chest compression. Further validation of the Intubrite®, Coopdech®, and Copilot® laryngoscopes in a clinical setting is required.

## Abbreviations

AHA: American Heart Association
CC: Chest compression
CPR: Cardiopulmonary resuscitation
EMS: Emergency medical services
ERC: European Resuscitation Council
ETI: Endotracheal intubation
ILCOR: International Liaison Committee on Resuscitation
